# Classroom injustice and university students’ cyberloafing: the mediating role of neutralization techniques

**DOI:** 10.3389/fpsyg.2025.1647669

**Published:** 2025-08-29

**Authors:** Zhiyong Han, Zhiyong Yang, Ziwei Huang

**Affiliations:** School of Business Administration, Anhui University of Finance and Economics, Bengbu, China

**Keywords:** classroom justice, cyberloafing, neutralization, condemnation of condemners, appeal to higher loyalties

## Abstract

**Introduction:**

Cyberloafing in the classroom has been linked to adverse educational outcomes, undermining students’ learning and frustrating instructors. From a neutralization perspective, students may justify deviant acts when they perceive injustice. This study examined how perceived classroom justice relates to students’ intention to cyberloaf and tested the mediating roles of two neutralization techniques: condemning the condemners and appealing to higher loyalties.

**Methods:**

We conducted a questionnaire study with 310 university students recruited from multiple universities in the central and eastern regions of China. Measures assessed perceived classroom justice, intention to cyberloaf, and the neutralization techniques of condemning the condemners and appealing to higher loyalties. We tested a mediation model linking classroom justice to cyberloafing intention through these neutralization techniques.

**Results:**

Perceived classroom justice was negatively associated with students’ intention to cyberloaf. Classroom justice also negatively predicted condemning the condemners and appealing to higher loyalties. Furthermore, both neutralization techniques mediated the relationship between classroom justice and intention to cyberloaf.

**Discussion:**

Findings suggest that higher levels of classroom justice may deter students’ cyberloafing partly by weakening justificatory neutralizations. These results highlight the importance of enhancing classroom justice to reduce cyberloafing behaviors. Educators are encouraged to adopt strategies that strengthen perceptions of fairness in classroom practices.

## 1 Introduction

The concept of cyberloafing was first introduced in the workplace as a voluntary use of an organization’s Internet resources by employees for non-work-related activities during official working hours (Lim, 2002). However, in an increasingly digitalized world, the Internet is no longer an exclusive tool for workplace employees for a long time, and it has become commonplace for students to engage in learning in the classroom using connected devices such as smartphones or laptops (Ragan et al., 2014). Furthermore, the phenomenon of cyberloafing has also become prevalent in the field of education. A significant proportion of students (more than 60%) have been observed to utilize their Internet devices during class, studying, or writing assignments to engage in activities unrelated to the subject matter, including browsing web messages, replying to messages, playing online games, watching online videos and web novels, and so on (Cutino and Nees, 2017; Miheliè et al., 2022). Established studies have demonstrated the detrimental impact of students’ cyberloafing behaviors on educational environments, including negative effects on students’ academic performance (Wu et al., 2018), sense of meaning in life (Li et al., 2022), attitudes toward learning and engagement (Heflin et al., 2017), and the finding that cyberloafing is significantly associated with academic procrastination among students (Margaretha et al., 2022). From the workplace to the field of education, from employees to students, the phenomenon of cyberloafing is no longer confined to the domain of higher education. As electronic devices proliferate in primary and secondary school classrooms, will these children, who are not yet adults and lack self-control, be able to recognize the dangers of cyberloafing and control their behavior? In light of the potential dangers of cyberloafing, it is essential to explore empirical studies that influence students’ intention to engage in this behavior.

However, existing studies have concentrated on employees’ cyberloafing behavior in the workplace, with limited research in the field of education (Alyahya and Alqahtani, 2022). Coskun and Gokcearslan (2019) reviewed 28 studies on cyberloafing in education in 2019 and found that there has been a gradual increase in scholarly articles on cyberloafing in the field of education in recent years. Coskun and Gokcearslan (2019) identified a extensive scope for future research on cyberloafing in the field of education. Furthermore, the majority of studies have concentrated on individual characteristics or outcome variables. The most frequently investigated variables are gender, grade level and Internet experience. In contrast, relatively few studies have focused on the motivation of cyberloafing behavior. These studies have found a few factors that can predictive of students’ cyberloafing behaviors or intentions to engage in such activities. These include consumerism (Sharma, 2020), escapism (Metin-Orta and Demirutku, 2022), and attitudes and habits (Soh et al., 2018). These findings provide a useful reference point for further research.

This study introduces classroom justice perceptions as a possible predictor variable, motivated by two considerations. Firstly, current research indicates that students’ perceived classroom justice is related to student variables such as motivation, classroom engagement, and enthusiasm for the subject matter. Conversely, students’ perceptions of classroom injustice can lead to negative emotions such as anger and resistance, as well as negative behaviors including negative learning, retaliation, and verbal aggression against the teacher, which may include cyberloafing, which is viewed as withdrawn behavior (Chory-Assad and Paulsel, 2004; Estaji and Zhaleh, 2022). Secondly, it should be noted that the perception of fairness has been demonstrated to act as an antecedent variable in predicting employees’ intention to engage in cyberloafing in the workplace. It can be posited that employees may engage in cyberloafing when they perceive organizational injustices in the context of their assignments, procedures, and interactions. This leads us to hypothesize that perceptions of fairness may similarly serve as a predictor of students’ intention to engage in cyberloafing in the classroom.

This research introduces two neutralization techniques to explain the mechanisms by which classroom justice influences students’ intention to cyberloaf. These are condemnation of the condemner and appeal to higher loyalties. Neutralization techniques are methods of justifying deviations or violations, demonstrating that the deviant behavior is acceptable (Lowry et al., 2016). The condemnation of the condemner is a form of neutralization whereby the individual who has committed the violation directs their attention toward the accuser. By directing attention away from their own deviant behavior and toward the teacher or classroom rules, students are able to shift the focus away from their own actions and toward those of the other person, making it easier to ignore their own faults (Matza and Sykes, 1957). The appeal to higher loyalties posited that the deviant behavior was undertaken in pursuit of a higher ideal. Students believe that they respond to their friends’ messages in class for the purpose of maintaining friendships, or play with their cell phones in class to avoid boredom. They consider these online loafing activities to be more important to them than listening to class (Sharma, 2020). The utilization of rationalization techniques enables students to justify the use of their connected devices in the classroom for activities unrelated to the content of the course.

In conclusion, our study constructed a model with classroom justice as the antecedent variable and two neutralization techniques (condemnation of condemners and appeal to higher loyalties) as the mediator variables to explain students’ intention to cyberloaf in the classroom, aiming to clarify whether students’ perception of classroom justice can influence their intention to cyberloaf and the mechanism of the effect. It is hoped that this study will be useful to educators in increasing attention to classroom fairness to reduce students’ cyberloafing behavior.

## 2 Literature review and hypothesis development

### 2.1 Theoretical framework

This study constructs a theoretical model based on neutralization theory to explain how perceptions of classroom justice influence university students’ cyberloafing behaviors through neutralization techniques. Neutralization theory explains how individuals use cognitive reframing to justify deviant behaviors, thereby reducing moral burden (Kaptein and van Helvoort, 2019). The theory posits that individuals are not inherently lacking in moral constraints; rather, they temporarily suspend such constraints through a series of “neutralization techniques,” making transgressive behaviors acceptable in specific contexts (Connolly et al., 2025). In educational settings, when students perceive a lack of classroom justice, they are motivated to employ various neutralization techniques to rationalize their negative learning behaviors (such as cyberloafing). Specifically, the theoretical logic of this study follows the following pathway: first, classroom justice, as an important characteristic of the learning environment, directly affects students’ learning motivation and behavioral choices. When students perceive distributive injustice, procedural injustice, or interactional injustice, they experience frustration and dissatisfaction; this perception of injustice constitutes the situational condition that activates neutralization techniques. Second, from the perspective of neutralization theory, perceived injustice prompts students to initiate cognitive defense mechanisms, namely employing neutralization techniques such as condemnation of condemners and appeal to higher loyalties to find legitimate justifications for their deviant behaviors. The application of these neutralization techniques reduces students’ moral concerns about cyberloafing behaviors, thereby increasing their willingness to engage in cyberloafing during class.

**FIGURE 1:**
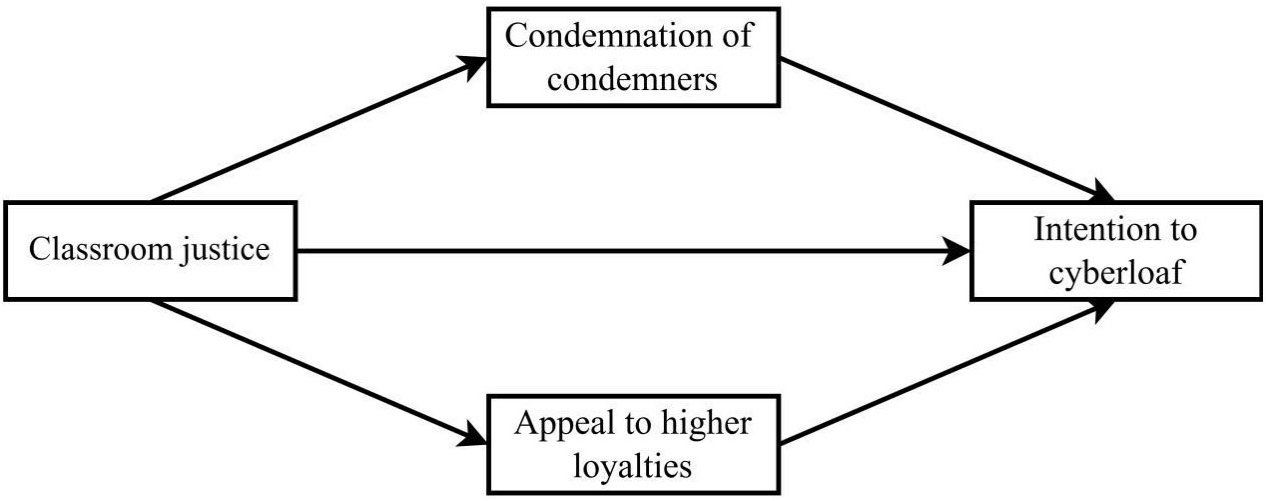
The conceptual research model.

Therefore, neutralization techniques played a significant mediating role between classroom justice perception and cyberloafing intention, constituting a theoretical transmission pathway from classroom justice perception to cyberloafing intention via neutralization techniques. This model not only extends the application of neutralization theory in educational contexts but also provides a new theoretical perspective for understanding university students’ classroom deviant behaviors in the digital age. The following section will systematically review related literature from three aspects and propose research hypotheses based on this foundation. Figure 1 presents the conceptual research model.

### 2.2 Classroom justice and the intention to cyberloaf

Chory-Assad (2002) defined classroom justice as the perception of fairness in the outcomes or processes that occur in an instructional setting (Chory-Assad, 2002). He initially categorized classroom justice into two dimensions: distributive justice and procedural justice. In subsequent research, he introduced the dimension of interactional justice, by which time the concept of classroom justice was relatively well developed. With regard to the interpretation of these three dimensions, distributive justice refers to perceptions of the fairness of the outcomes distributed in an educational setting (e.g., grades, teacher attention), procedural justice refers to the fairness of the processes used to assign grades (Chory-Assad and Paulsel, 2004). Interactional justice is defined as the perceived fairness of the teacher’s interpersonal treatment of students when performing a process or assigning an outcome (Chory, 2007). Classroom injustice is a pervasive phenomenon, with numerous students reporting that they have experienced unfair treatment from teachers in the classroom. Research indicates that students suffer from almost three times as much procedural injustice as the other two forms of injustice (Horan et al., 2010; Yan, 2021). Students’ perceptions of classroom justice affect their subsequent behaviors and emotions, and have been shown to correlate with a multitude of student variables, including students’ academic performance, willingness to speak up, classroom participation, and teacher-student relationships. As early as Chory-Assad (2002) demonstrated that classroom justice influenced students’ motivation to succeed in the course, course affective learning, and aggression toward the teacher. Furthermore, Chory found that procedural justice negatively predicted retaliatory and deceptive behaviors (Chory-Assad, 2002, 2004). The study conducted by Čiuladienė and Račelytė (2016) demonstrated a correlation between students’ perceptions of unfairness in classroom environments and the manifestation of negative behaviors, including fighting, dishonesty, anger, and struggles. In a recent study, Chory (2023) reported that college students’ perceptions of fairness were related to antisocial classroom behaviors. A substantial body of research has shown that unfairness in the classroom is significantly associated with students’ negative behaviors and emotions. Consequently, it can be posited that classroom justice predicts cyberloafing activities, which also serve as negative behaviors.

Our suggestion that classroom justice relates to intention to cyberloaf is not without empirical support. In organizational contexts, perceptions of fairness have been demonstrated to be an effective predictor of employees’ cyberloafing behavior (Jandaghi et al., 2015). When employees employ a technique of ledger metaphors to justify subsequent cyberloafing in the context of perceived organizational injustice (Lim, 2002). Subsequent research has also demonstrated a close relationship between perceptions of justice and cyberloafing (Samadi-Miarkolaei and Samadi-Miarkolaei, 2020). When this result was considered in the context of educational, the Yılmaz and Yurdugül (2018) study found that organizational policies and positive organizational equity management in the classroom positively affect the classroom environment, which reduces cyberloafing. Nevertheless, Yılmaz and Yurdugül (2018) concentrated on the psychosocial environment, and further in-depth discussion is required regarding the predictive mechanism of classroom equity as a separate variable on students’ cyberloafing behavior.

Furthermore, equity theory posits that individuals confronted with an unjust situation are driven to take action to rectify the imbalance, with these actions including reducing personal inputs to levels commensurate with the rewards received or attempting to evoke a similar sense of resentment in those responsible for the injustice (Sprecher, 1986; Sprecher and Felmlee, 1992). Building on this theoretical foundation, we propose that when students perceive that the effort they put into the course is not rewarded with a corresponding grade or that their behaviors and ideas are not respected by the instructor, feelings of injustice emerge, which in turn can lead to negative feelings of anger and resistance to the instructor (Estaji and Zhaleh, 2022). This further enhances students’ willingness to engage in cyberloafing activities. Students express their dissatisfaction by engaging in cyberloafing activities that are unrelated to the course content, thereby resisting the injustice that exists in the classroom. It is therefore proposed that the following hypothesis be tested:

H1: Classroom justice is negatively related to students’ intention to cyberloaf.

### 2.3 Classroom justice and two neutralization techniques

Matza and Sykes (1957) proposed that juvenile crime is the result of a neutralization technique, which allows individuals to justify their deviant or criminal behavior. In particular, when adolescents engage in behavior that violates societal rules, they tend to experience shame and self-blame. This shame inhibits most deviance. In order to alleviate this negativity, they convince themselves that they have not done anything wrong through the use of words that allow them to engage in criminal behavior with a sense of peace of mind. “I did not cause any harm,” “I did not act for my own benefit,” “It is not my responsibility.” By invoking these excuses, individuals are able to justify their behavior in a manner that is contrary to the values and societal norms that they espouse. This enables them to preserve their self-image and avoid self-blame and the blame of others for deviant behavior. Matza and Sykes (1957) categorize such “neutralization” into five distinct categories: denial of responsibility, denial of injury, denial of victimization, condemnation of the condemner, and appeal to higher loyalties. In subsequent studies, scholars have further developed the neutralization technique by proposing the metaphor of the ledger (Klockars, 1974) and the defense of necessity (Minor, 1981), among other techniques. Sykes posits that the utilization of this “rationalization technique” is evidence-based. Given that social norms are malleable, perpetrators can circumvent social sanctions if they are able to provide a rationale for their actions that is deemed acceptable by the public. For instance, the killing of an enemy during wartime is permitted by public perception, and justifications such as the killing of minors, the insane, or in self-defense are also provided for in the law. It is unfortunate that the majority of transgressions are not acceptable to the law and society for their own narrow reasons. Neutralization techniques are a common feature of criminological studies. Murphy and Dacin (2011), in their investigation into the prevention of fraud, found that those who engage in fraudulent behavior employ neutralization techniques to avoid or minimize the negative consequences of their actions. Similarly, In their research on the psychology of tax evaders and welfare fraudsters, Marriott and Lai found that neutralization theory was widely used by both types of offenders to excuse their illegal behaviors (Marriott and Lai, 2023). A number of other studies have also identified the use of neutralization techniques in relation to deviant behaviors. These include the subculture of right-wing violence (Colvin and Pisoiu, 2021), white-collar crime (McGrath, 2021), and workplace cyber-abuse (van Baak et al., 2023).

It is inevitable that while neutralization theory is widely supported as a framework for understanding violations, it remains deficient in some respects (Maruna and Copes, 2005). McLean K points out that there is no discussion in Matza and Sykes’s (1957) study of how or why neutralization occurs, and that the mechanisms by which individuals engage in neutralization techniques are ambiguous. He further suggests that perceptions of procedural injustices may be one of the sources of neutralization (McLean and Wolfe, 2016). Accordingly, this study broadens its scope to encompass the perception of fairness as a potential factor influencing the efficacy of neutralization techniques.

Previous research has provided a foundation for the construction of a relationship between perceptions of classroom justice and neutralization techniques. In his 1964 refinement of Sykes’s theory of neutralization, Matza observed that whenever a sense of injustice is prevalent, the moral restraints of the law are loosened, allowing neutralization techniques to take advantage of the situation. This suggests that perceptions of injustice are the forerunners of neutralization techniques (Matza, 2018). Further studies have demonstrated a correlation between procedural justice and neutralization techniques. These studies have indicated that perceptions of procedural injustice can lead to a relaxation of moral constraints, and that individuals are more likely to adopt neutralization techniques as a result (McLean and Wolfe, 2016; Lazarus et al., 2025). The aforementioned studies confirm that there is indeed a link between fairness perceptions and neutralization techniques. However, following a search and organization of the literature, no empirical studies have been found that examine how students’ perceptions of fairness affect their use of neutralization techniques in an educational setting. Consequently, the present study sought to establish a link between perceptions of classroom justice and the use of neutralization techniques.

The perceptions of students regarding justice can influence the utilization of the condemnation of condemners’ technique. The condemnation of condemners is a process whereby a person who has committed a transgressive act subsequently attacks the target (either a person or an object) who has blamed them (Byers et al., 1999). Matza and Sykes (1957) note that by attacking others, perpetrators divert attention away from their own transgressive behavior to the motives and actions of those opposing their actions, making it easier for them to overlook their own faults. In classroom contexts, the specific manifestations of students using the condemnation of condemners technique to justify their cyberloafing behaviors include: when caught using mobile phones during class, students might say “The teacher’s lecture is too boring, just reading from PPT slides, of course I need to find something else to do” (questioning the teacher’s teaching competence); “This teacher obviously favors good students and doesn’t care about ordinary students like us, why should I listen attentively” (questioning the teacher’s fairness) (Chory, 2023). Students will find reasons to excuse their own transgressive behavior, such as complaining about a teacher’s poor teaching methods or poor classroom organization (Alexander and Opsal, 2021; Walsh et al., 2021). The perception of unfairness provides another good excuse for students to condemn their teachers for the purpose of transferring blame. Students who perceive that they have been treated unfairly exhibit more hostility and aggression toward their teachers, and an increase in retaliatory and deceptive behaviors (Chory et al., 2017). They are reluctant to interact or talk in class and exhibit negative classroom emotions (Horan et al., 2012). Furthermore, research indicates that students’ consumerist tendencies result in them consistently identifying areas of dissatisfaction with their educators (Sharma, 2020). Since teachers serve as authority figures in the classroom system, their competence, character, and care largely determine the level of classroom justice (Chory, 2007). When students perceive that they are treated unfairly in the classroom, such as through unfair grading, unfair rewards and punishments, or unequal teaching resources, they will direct their frustration toward the teacher, developing resentment toward him or her. Students’ perceptions of injustice in the classroom elicit emotional and behavioral responses, the most overt of which are manifestations of anger and dissent. The perception of unfair treatment by the teacher leads to a decline in the teacher’s authority and respect among students. This, in turn, results in students denouncing the teacher’s unfairness to themselves, their classmates, and even the next students. Consequently, we put forth the following hypothesis:

H2: Classroom justice is negatively related to the condemnation of condemners.

Another manifestation of students’ perceptions of classroom justice is the appeal to higher loyalties. This refers to individuals justifying their deviance behavior by claiming that it is in pursuit of higher levels of value and meaning (Li and Cheng, 2013). The core of this technique lies in that individuals do not deny that their behavior may have violated certain rules, but claim that such behavior stems from loyalty to more important values or goals (Alexander and Opsal, 2021). Sykes notes that offenders are caught in a dilemma, having to choose between special needs and breaking the rules. They do not deny that their behavior violates the rules of mainstream society; however, they believe that it is justified to break the rules for a more meaningful purpose, such as for friendship (Matza and Sykes, 1957). A similar phenomenon can be observed in the classroom setting, when browsing web pages or using social media during class, students might justify their behavior by saying “I’m searching for the latest materials related to this course; online information is more timely than textbooks” (claiming to pursue more current knowledge); “I’m preparing for upcoming job hunting; submitting resumes is more helpful for my future than listening to these theoretical courses” (emphasizing loyalty to personal development) (Čiuladienė and Račelytė, 2016; Chory, 2023). Students believe that they are now engaging in activities in the classroom that are not related to course content, such as completing assignments for other courses, responding to messages from instructors or friends, or even taking breaks, is more important than listening to lectures. Consequently, students are compelled to choose between their allegiance to the classroom and their engagement with non-classroom activities. Contemporary students exhibit a stronger claim to equality and justice in the classroom than their predecessors. They feel that they can choose to participate or withdraw from the classroom based on how they feel (Pathan et al., 2017). For students who perceive injustice in the classroom, the value of the classroom and the authority of the teacher are diminished, and compliance with classroom rules appears to become less important. The fostering of such feelings of injustice encourages students to cease demonstrating loyalty to the classroom, thereby shifting the balance of loyalty toward activities that are not directly related to the classroom. Consequently, we put forth the following hypothesis:

H3: Classroom justice is negatively related to appeal to higher loyalties.

### 2.4 Two neutralization techniques as mediators

The term “classroom cyberloafing” refers to the online deviant behavior of students who use the Internet and mobile devices in the classroom to engage in activities unrelated to the learning content, such as browsing entertainment news, watching online videos or sending messages in the classroom (Kalaycı, 2010). A number of studies have demonstrated that students’ engagement in cyberloafing in the classroom has a detrimental impact on education. This not only reduces students’ classroom engagement but also has a negative effect on teachers’ enthusiasm and effectiveness (Gerow et al., 2010). Upon realizing the potential dangers of cyberloafing, scholars began to rely on various theories in order to identify factors that could explain cyberloafing from multiple perspectives. For instance, Gerow, employing a field theory approach that considers both external and internal forces, identified that social norms (beliefs about rules), cognitive absorption (the state of being deeply engaged with online technology in the classroom), and multitasking (the ability to perform multiple tasks simultaneously) all have an impact on students’ intentions to engage in cyberloafing (Gerow et al., 2010). Taneja et al. (2015) also investigated the potential influence of various factors on students’ intentions to utilize connected devices in the classroom. These include consumerism, escapism, attention deficit, cyberloafing anxiety, and distraction from others’ cyberloafing behaviors (Taneja et al., 2015). Furthermore, scholars have employed the theoretical lens of the theory of planned behavior to demonstrate that both habit and intention are significantly associated with cyberloafing behavior (Soh et al., 2018). The investigation into student cyberloafing is ongoing, and the application of neutralization techniques offers a distinctive approach to furthering the research. The neutralization technique places greater emphasis on the human heart and considers student cyberloafing within the broader context of criminal behavior, in order to gain insight into the perceived “freedom” of students.

Neutralization techniques can be a significant predictor of intention to engage in cyberloafing. In organizational contexts, when individuals perceive that the remuneration they receive is not commensurate with the quantity of work they perform, they utilize a form of ledger-like neutralization technique to justify their subsequent cyberloafing behavior (Lim, 2002). Some studies have demonstrated that employees utilize a multitude of neutralization techniques to justify their cyberloafing behavior in the workplace (Batabyal and Bhal, 2020). The explanatory role of neutralization theory is equally applicable in the field of education. Sharma examined the impact of neutralization techniques and consumerism on students’ intention to cyberloaf in the classroom, which is one of the few studies to apply neutralization theory to students’ cyberloafing (Sharma, 2020). Nevertheless, the mechanism of action of neutralization techniques as a pathway to explore the relationship between classroom justice and intention to cyberloaf has not yet been studied.

The present study proposes that classroom justice negatively predicts students’ intention to engage in cyberloafing through condemnation of condemners. This is based on the following reasons. Firstly, students’ perceptions of classroom injustice can result in dissent and dissatisfaction with the teacher, which in turn can positively affect the condemnation of condemners technique. Secondly, it is our contention that the condemnation of condemners will result in an increase in students’ willingness to engage in cyberloafing. Individuals have an innate desire to present a positive image of themselves, and most offenders are able to recognize that their behavior is wrong, creating a conflict between the need to maintain an image and transgressive behavior (Matza and Sykes, 1957; Robinson and Kraatz, 1998). Thus, by denouncing the denouncers, a solution to this internal conflict can be found. In particular, by criticizing or complaining about their teachers, students are able to deflect the blame and attention away from themselves, thereby reducing the shame and guilt they feel as a result of engaging in cyberloafing behavior. Students who engage in cyberloafing may attribute their actions to unreasonable classroom rules or instructional content that fails to engage them, despite the time they are investing in the activity. Therefore, it is not appropriate to hold students responsible for engaging in cyberloafing, which is the responsibility of the teacher and the school. Previous research has also confirmed the significant positive predictive effect of condemnation of condemners’ technique on the intention to cyberloaf (Li and Cheng, 2013; Gügerçin, 2020). As a result, we argue that students who feel injustice in the classroom have no qualms about engaging in cyberloafing by shifting the blame and fault to others through the condemnation condemner technique. Therefore, we propose:

H4: Condemnation of the condemners mediates the negative relationship between classroom justice and intention to cyberloaf.

The appeal to higher loyalties plays a similar role. The perception of injustice has a detrimental effect on the teacher-student relationship, as well as the authority of classroom rules and the teacher, resulting in a decline in classroom loyalty and an increase in students’ intention to engage in cyberloafing. Individuals are always evaluating the advantages and disadvantages of a given situation and tend to select the course of action that will yield the greatest benefit (Homans, 1961). Students who engage in behaviors unrelated to the content of the class claim that their cyberloafing behavior is driven by the pursuit of higher expectations, the desire to recover from the experience, the avoidance of boredom, or the need to fall asleep in class. The experience of injustice made the classroom less meaningful and demonstrated a lack of loyalty to the classroom. Research indicates that students who exhibit a lack of loyalty to the classroom are more likely to engage in cyberloafing (Sharma, 2020). Students with less loyalty to the classroom are more likely than students with more loyalty to the classroom to engage in behaviors that violate classroom rules, such as responding to a friend’s message or completing other coursework during class. Furthermore, cyberloafing can be conceptualized as a form of withdrawal behavior, reflecting an individual’s attempts to cope with dissatisfaction and perceived injustice (Askew, 2012; Askew et al., 2014). Consequently, we posit that injustice prompts a decline in classroom loyalty, prompting students to seek out alternative sources of attachment and increasing their likelihood of engaging in cyberloafing. Consequently, we put forth the following hypothesis:

H5: Appeal to higher loyalties mediates the negative relationship between classroom justice and intention to cyberloaf.

## 3 Research methods

### 3.1 Sample and method

Prior to commencing the data collection process, the questionnaire underwent a preliminary testing phase with 20 undergraduate students. As the questionnaire was translated from a mature foreign questionnaire, it was necessary to make minor modifications to the word order and wording of some items in order to avoid comprehension bias. These modifications were based on the comments and feedback from the 20 respondents after they had completed the questionnaire.

This study employed a two-stage survey design with stratified sampling methodology to investigate 310 university students from various universities in the central and eastern regions of China between March and May 2024. Considering that regional differences, university types, and academic backgrounds might influence students’ perceptions of classroom justice and online usage behaviors, we conducted systematic sampling through the user pool of “Credamo,” a professional online survey platform. This platform represents one of China’s largest professional online survey platforms, covering students from universities across multiple regions and levels nationwide, thereby providing an excellent sampling framework for this research. In terms of sampling design, we primarily focused on higher education institutions in East China and Central China regions, including both key universities and regular university institutions of different tiers. The sample encompassed major disciplinary categories such as economics and management, liberal arts and philosophy, and science and engineering, as well as students from freshman to senior years and graduate students at various levels, ensuring sample diversity and representativeness. The specific sampling procedure consisted of three stages. First, screening criteria were established within the platform’s user pool, including active university student status, age range of 18–25 years, and residence in target regions, initially screening approximately 550 potential participants who met these requirements. Second, to ensure reasonable sample structure, we established quotas for various strata, including gender proportion, uniform distribution across different academic categories, and relatively balanced representation across grade levels. Finally, survey invitations were randomly distributed among qualified users following a first-come-first-served principle until the predetermined sample size was achieved. Participants completed the questionnaire online and received compensation of 2 RMB. Throughout this process, strict quality control measures were implemented, including IP address restrictions to prevent duplicate responses, response time monitoring, attention check items, and detection of consistent response patterns to eliminate obviously careless responses. Through this systematic sampling procedure, the final sample successfully achieved the expected diversity requirements in terms of regional distribution, institutional types, academic composition, and grade level distribution. This study collected data in two stages, in accordance with the recommendations set forth by Podsakoff et al. (2003). In the initial phase, data pertaining to demographic characteristics, perceptions of classroom justice, and two mediating variables were collected. The intention to engage in cyberloafing was measured one month apart in the second stage. In the initial phase, 366 questionnaires were distributed, and 331 valid responses were obtained, representing a 90.44% recovery rate. In the subsequent phase, 331 questionnaires were distributed, and 310 valid responses were collected, resulting in a 93.66% recovery rate. The aforementioned invalid responses were discarded due to the questionnaires being too brief to be completed or due to inconsistency in the responses or the absence of some responses.

A total of 310 valid questionnaires were obtained for the test of this study, of which 109 (35.2%) were males and 201 (64.8%) were females. The students who participated in the questionnaires were from different majors, including Economics and Management (34.5%), Literature, History, and Philosophy (22.3%), Science and Technology (36.1%), and Others (7.1%). The respondents had different levels of education, with approximately 85% of respondents studying at the undergraduate level and approximately 15% at the graduate level.

### 3.2 Measurement tools

The online questionnaire comprised four sections: the Classroom Justice Scale, the Neutralization Technology Scale, the Intention to Cyberloaf Scale, and demographic information. Students were asked to rate the items in the aforementioned questionnaire based on their true feelings. Responses to all variables were categorized on a five-point scale from strongly disagree (1) to strongly agree (5). The specific sources and content of each scale are listed below:

#### 3.2.1 Classroom justice

The measures of classroom justice comprise the distributive and procedural justice scales developed by Chory-Assad and Paulsel (2004) and the interactive justice scale developed by Chory (2007). The items were related to students’ perceptions of fairness in earning grades in the course, perceptions of fairness in evaluating instructor placement and course policies in the classroom, and perceptions of fairness in instructor-student interpersonal interactions. A total of 30 items were measured, including the following: “The way the instructor grades course grades,” and “The way the instructor treats students.” Cronbach’s alpha for the scale was α = 0.90.

#### 3.2.2 Two neutralization techniques

A maturation scale adapted by Sharma (2020) was employed to assess the two neutralization techniques of Condemnation of Condemners and Appeal to Higher Loyalties. Each of the two concepts was measured with three items, for example, “If the course policy is too strict, then it is not wrong to use the internet/smartphone/computer in violation of the course policy,” and “If you complete the tasks assigned by the instructor, then using the internet/smartphone/computer in the classroom is acceptable.” Cronbach’s alpha for these two scales was α = 0.80 and α = 0.86.

#### 3.2.3 Intention to cyberloaf

The scale developed by Taneja et al. (2015) was employed to assess students’ intentions to engage in cyberloafing, which was also measured with three items, such as “I plan to use the Internet/mobile phone for non-classroom related activities in future classes.” Cronbach’s alpha for the scale was α = 0.89.

#### 3.2.4 Control variables

It has been demonstrated by previous research that demographic variables, such as gender and grade level, influence students’ cyberloafing behavior (Fallows, 2005; Baturay and Toker, 2015; Knight, 2017). For instance, Baturay’s and Toker (2015) study revealed that male students exhibited a greater tendency to engage in cyberloafing than their female counterparts. Similarly, grade level was found to have an impact on online cyberloafing, with students in higher grades being more experienced and aware of classroom styles than those in lower grades, who demonstrated more intention to cyberloaf. Consequently, this study employed the gender variable, the grade level variable, and the education level as control variables.

## 4 Data analysis and results

### 4.1 Common method bias test

As all questions in this questionnaire were completed by subjects at the same time by self-report, which may lead to changes in the dependent variable that are derived from the same measurements rather than the effect of the independent variable. To test for common method bias, Harman’s single factor test was used (Podsakoff et al., 2003). We entered all questions into an unrotated principal component factor analysis and the results showed that factors with eigenvalues greater than 1 were extracted and the maximum factor variance explained was 21.48% (less than 40%). Therefore, we can conclude that there is no significant common method bias in this study and its effect on the final measures is minimal.

### 4.2 Confirmatory factor analysis

Before validating the structural model, we need to evaluate the measurement model. The evaluation of the measurement model includes reliability, structural validity, convergent validity and discriminant validity, and in this study, these criteria were examined using SPSS 26.0 and AMOS software. As shown in Table 1, Cronbach’s alpha (α), composite reliability (CR), average variance extracted (AVE), maximum shared variance (MSV) and square root of AVE values are presented. An acceptable Cronbach alpha index (α > 0.70) was obtained for all variables in this study, indicating that the scale expressed a satisfactory level of reliability. For structural validity, the goodness of fit indices [χ^2^ (310) = 98.915, χ^2^/df = 2.061, IFI = 0.973, TLI = 0.963, CFI = 0.973, RMSEA = 0.059] were within reasonable limits, supporting the plausibility of the hypothesized model. Secondly, discriminant validity was examined by comparing the magnitude of the square root of AVE with the correlation coefficients between the variables. The values of the square root of AVE for the four variables were greater than their correlation coefficients and MSV < AVE, indicating that the discriminant validity of the scale was good. Finally, we examined whether the composite reliability (CR > 0.7) and average variance extracted (AVE > 0.5) were within reasonable intervals, and the factor loadings of each question item were greater than 0.5, indicating that the convergent validity was ideal, as shown in Table 1.

**TABLE 1:** Reliability and validity.

Construct	1	2	3	4	α	CR	AVE	MSV
1. Classroom justice	**0.744**				0.902	0.781	0.553	0.105
2. Condemnation of condemners	−0.190	**0.758**			0.799	0.801	0.574	0.497
3. Appeal to higher loyalties	−0.222	0.705	**0.819**		0.857	0.859	0.671	0.516
4. Intention to cyberloaf	−0.324	0.563	0.718	**0.858**	0.892	0.894	0.737	0.516

*N* = 310; MSV, maximum shared variance; Bolded values on the diagonals of columns 2–5 are the square root values of AVE.

### 4.3 Correlation analysis

The means and correlations of the variables are presented in Table 2. The correlation analysis results show that classroom justice exhibits a weak negative correlation with condemnation of condemners (*r* = −0.143, *p* < 0.05) and with appeal to higher loyalties (*r* = −0.174, *p* < 0.01), and a moderate negative correlation with cyberloafing (*r* = −0.278, *p* < 0.01). These negative correlations indicate that the higher students’ perception of classroom justice, the lower their tendency to use neutralization techniques and engage in cyberloafing. Notably, the two neutralization techniques show a moderate positive correlation (*r* = 0.594, *p* < 0.01), suggesting that students tend to use multiple neutralization techniques simultaneously. Condemnation of condemners exhibits a moderate positive correlation with cyberloafing (*r* = 0.485, *p* < 0.01), while appeal to higher loyalties shows a strong positive correlation with cyberloafing (*r* = 0.626, *p* < 0.01), indicating that the use of neutralization techniques is closely related to cyberloafing intentions, with appeal to higher loyalties showing a stronger association with cyberloafing. These correlational relationships provide preliminary support for our research hypotheses and establish the foundation for subsequent regression analysis and mediation effect testing.

**TABLE 2:** Means and correlations.

Construct	Means	SD	1	2	3
1. Classroom justice	3.93	0.42			
2. Condemnation of condemners	2.55	0.88	−0.143*		
3. Appeal to higher loyalties	3.44	0.96	−0.174**	0.594**	
4. Intention to cyberloaf	2.99	1.05	−0.278**	0.485**	0.626**

*N* = 310, **p < 0.01,*p < 0.05.

### 4.4 Testing of hypotheses

In this study, Hayes’ (2013) SPSS Extended Macro-process Model 4 (a simple mediation model) was used to test the mediating role of condemnation of condemners and appeal to higher loyalties in the relationship between classroom justice and intention to cyberloaf, and the other hypotheses mentioned above were tested by regression analysis. The results of the tests are shown in Table 3: as can be seen from the results of the tests of Models 2 and 3, students’ perceptions of classroom justice are significant predictors of condemnation of condemners (β = −0.130, *p* < 0.05) and appeal to higher loyalties (β = −0.153, *p* < 0.01); therefore, Hypotheses 2 and 3 are verified. Classroom justice was a significant predictor of intention to cyberloaf (β = −0.255, *p* < 0.01) and the value of the direct effect of perceived classroom justice on intention to cyberloaf was −0.39 (SE = 0.11, 95% IC [−0.6, −0.18]) (Table 4), which is a significant direct effect; Hypothesis 1 was verified. In addition, from the test results of Model 5, it can be seen that condemnation of condemners (β = 0.162, *p* < 0.01) and appeal to higher loyalties (β = 0.491, *p* < 0.01) are also significant predictors of the intention to cyberloaf.

**TABLE 3:** Regression results.

Construct	Condemnation of condemners		Appeal to higher loyalties	Intention to cyberloaf	
	Model 1	Model 2	Model 3	Model 4	Model 5
Gender	0.137	0.063	0.112	0.101	0.036
Educational level	−0.06	−0.007	−0.125	−0.055	0.007
Major	−0.149	−0.053	−0.076	−0.136	−0.09
Classroom justice		−0.130*	−0.153**	−0.255**	−0.158**
Condemnation of condemners					0.162**
Appeal to higher loyalties					0.491**
R^2^	0.045	0.028	0.038	0.109	0.449
△R^2^	0.045	0.017	0.038	0.063	0.149
F	4.859**	2.164*	4.952**	9.304**	41.226**

*N* = 310, **p < 0.01,*p < 0.05.

**TABLE 4:** Direct and indirect effects and 95% confidence intervals.

Effect	B	SE	*t*-value	LL	UL
**Direct effect**					
Classroom justice→intention to cyberloaf	−0.39	0.11	−3.6	−0.60	−0.18
**Indirect effects**					
Classroom justice→condemnation of condemners→intention to cyberloaf	−0.05	0.03		−0.13	−0.002
Classroom justice→appeal to higher loyalties→intention to cyberloaf	−0.19	0.07		−0.33	−0.06

*N* = 310; B, unstandardized regression coefficients; SE, standard error; Bootstrap sample size = 5,000; LL, lower limit; UL, upper limit.

Bootstrap was used to further test the mediating effects of the two neutralization techniques, and classroom justice had a significant indirect effect on students’ intention to cyberloaf through condemnation of condemners (*B* = −0.05, LLCI = −0.13, ULCI = −0.002), LLCI (Lower Level Confidence Interval) represents the lower bound of the confidence interval, while ULCI (Upper Level Confidence Interval) represents the upper bound of the confidence interval. Since the 95% confidence interval does not contain 0 (ranging from −0.13 to −0.002, all negative values), this indicates that the indirect mediation effect of condemnation of condemners is statistically significant, providing support for Hypothesis 4. Classroom justice had a significant indirect effect on students’ intention to cyberloaf by appealing to higher loyalties (*B* = −0.19, LLCI = −0.33, ULCI = −0.06). Similarly, since the confidence interval [−0.33, −0.06] does not contain 0, this indicates that the indirect mediation effect of appeal to higher loyalties is statistically significant, providing support for Hypothesis 5.

## 5 Discussion

Does students’ perceived classroom justice predict their intention to engage in cyberloafing in the classroom? Existing research provides some theoretical basis for addressing this question. On the one hand, the current study confirms that organizational justice, also a fairness variable, significantly predicts employees’ intentions to engage in cyberloafing in the workplace; on the other hand, the fact that classroom justice are significantly associated with deviant behaviors is also validated. Based on the neutralization theory, this study introduces two neutralization techniques, condemnation of condemners and appeal to higher loyalties, as mediating variables to explore the effects of classroom justice on the intention to engage in cyberloafing and the mechanisms involved by sampling college and university students from different regions of China. The results of the study show that, first, we found that students’ perceived classroom justice was negatively related to their intention to cyberloaf (β = −0.255, *p* < 0.01), and this moderate effect holds important practical managerial value. This effect size indicates that, compared to technological restrictions or punitive measures, enhancing classroom justice represents a highly cost-effective strategy for reducing student cyberloafing. For educators, this means that by establishing transparent grading criteria and ensuring equal opportunities for classroom participation, student cyberloafing behaviors can be significantly reduced. This finding is consistent with conclusions from previous theoretical research, when students perceive themselves to be treated fairly, they are more likely to follow school rules and regulations (Rasooli et al., 2022). However, a not insignificant number of students report that they have experienced unfair treatment by their teachers (Smith and Gorard, 2012), and these students are more likely to engage in deviant behaviors (Morris, 2010), which may include cyberloafing. Students seek to restore justice by engaging in cyberloafing behaviors in the classroom that are unrelated to the content in order to retaliate or boycott their teachers.

Second, our study found that students’ perceived classroom justice was negatively correlated with both condemnation the condemners and appeal to higher loyalties. Notably, the effect of classroom justice on appeal to higher loyalties (β = −0.153) is slightly stronger than its effect on condemnation of condemners (β = −0.130), with a higher level of significance. This suggests that when students face classroom injustice, they are more inclined to rationalize their behavior by seeking “more valuable” alternative activities rather than directly condemning teachers. Teachers are in control of the classroom and student-teacher conflicts are also originally common (Čiuladienė and Račelytė, 2016). When students perceive that they are treated unfairly by their teachers, they naturally make the teacher the object of their condemnation, they blame, resist and even attack the teacher (Chory-Assad and Paulsel, 2004). Students perceive that they are not being respected by their teachers and they are equally justified in ceasing to be loyal to teachers. Their loss of interest in classroom content motivates them to do things they find more meaningful, including responding to messages or completing assignments for other courses, which they see as more important than listening to lectures (Zito and McQuillan, 2010). We further confirmed that neutralization techniques are positively associated with students’ intention to cyberloaf, a result that remains largely consistent with the findings of most studies.

Finally, our study shows that students’ perceived classroom justice influences their intention to cyberloaf through two paths: condemnation of condemners and appeal to higher loyalties. More importantly, we found that the two mediation pathways confirmed by our research demonstrate different effect magnitudes, with the indirect effect of the appeal to higher loyalties pathway (*B* = −0.19) being nearly four times stronger than that of the condemnation of condemners pathway (*B* = −0.05), providing prioritized guidance for tiered interventions. Based on the stronger mediation effect of appeal to higher loyalties, priority should be given to enhancing the attractiveness of courses and classroom activities, including improving the intrinsic value of courses, creating immersive learning experiences, and helping students establish personalized learning goals. Based on the smaller but significant mediation effect of condemnation of condemners, this should be treated as a supplementary strategy. From the perspective of theoretical mechanisms, neutralizing technology is an excellent factor in explaining students’ use of the Internet in the classroom for activities unrelated to classroom content. Students’ perceived unfairness in the classroom served as a good precondition for students to use neutralization techniques to justify their cyberloafing behavior. When teachers are unfair in assigning grades, rewards, or punishments, students are likely to invoke neutralization techniques to justify the infractions they subsequently engage in. The ability of the neutralization technique to alleviate their feelings of guilt led students to develop a higher willingness to cyberloaf. Some student comments support these judgments, “Teachers give pretty much higher grades to all students, in order to gain general student approval and goodwill. The effort and seriousness you show in such a class is useless, and there is little difference in your final grades between you who actively answer questions and the students who are absent and leave early or who surf the net on their cell phones in class.” “It’s just not fair that some courses have outdated content that the teacher will just read from a PowerPoint, while I have to spend all my time and energy to take in this outdated knowledge.” Students neutralize this sense of injustice by denouncing the teacher or engaging in something they find more meaningful than listening to a lecture, causing them to show a greater intention to cyberloaf.

### 5.1 Theoretical contributions

First, the outstanding theoretical contribution of this study is the introduction of classroom justice variables to explain students’ intention to cyberloaf, expanding the research on factors influencing intention to engage in cyberloaf behaviors under the domain of education. Fairness is one of the important variables in explaining transgressions, and people’s perception of unfairness is closely linked to anger, revenge, etc (Muhammad et al., 2024). Lim (2002) achieved good results by using employees’ perceived fairness in the organization as an antecedent variable to explain employees’ cyberloafing behavior in the workplace. In addition, existing research has confirmed that the link between perceived unfairness and transgressive behavior holds true for students as well (Chory, 2023). However, there is little research on the link between classroom justice and intention to cyberloaf, and our study is expected to make a contribution in this regard. The results of this study enrich the research on factors influencing students’ cyberloafing behaviors in classroom settings and help promote a more comprehensive picture of cyberloafing research.

Second, the theoretical contribution of this study lies in applying neutralization theory to examine the relationship between classroom justice and cyberloafing, expanding the application scope of neutralization theory in educational contexts. Since Matza and Sykes (1957) proposed neutralization theory, it has primarily been applied to explain traditional criminological domains such as juvenile delinquency and white-collar crime. Although scholars have recently extended it to organizational behavior and applied it to explain students’ deviant behaviors and cyberloafing, research specifically examining classroom justice as an antecedent variable to explore its impact on cyberloafing through neutralization techniques remains relatively limited (Sharma, 2020; Ainsworth et al., 2024). Our study further deepens theoretical exploration in this field based on existing foundations, validates the applicability and explanatory power of neutralization theory in educational environments, and demonstrates the theory’s universal value beyond traditional criminological domains. The innovative significance of this cross-disciplinary theoretical expansion manifests in three levels: expansion of theoretical boundaries—proving that neutralization techniques apply not only to major deviant behaviors but also to minor deviant behaviors in educational contexts; enrichment of conceptual connotations—discovering unique manifestations of neutralization techniques in educational contexts, such as students redefining learning value through “appeal to higher loyalties” and questioning educational authority through “condemnation of condemners”; deepening of theoretical mechanisms—revealing differentiated operational patterns of neutralization techniques in educational contexts, where the mediation effect of appeal to higher loyalties is significantly stronger than that of condemnation of condemners, indicating that students are more inclined to handle perceived injustice by reconstructing behavioral meaning rather than direct confrontation.

Moreover, the core theoretical contribution of this study lies in constructing a complete explanatory framework wherein classroom justice influences cyberloafing via neutralization techniques. On one hand, we introduced classroom justice into cyberloafing research and confirmed the significant negative predictive effect of classroom justice on students’ cyberloafing intentions. On the other hand, we discovered that classroom injustice provides cognitive foundations for students to activate neutralization techniques and validated the crucial mediating role of neutralization techniques in this process. This contrasts sharply with previous cyberloafing research that primarily focused on conservation of resources theory and theory of planned behavior, providing a novel theoretical perspective for understanding the social psychological mechanisms of cyberloafing (Chavan et al., 2022; Zhou et al., 2023). Particularly noteworthy is our discovery of unique operational patterns of neutralization techniques in educational contexts—“appeal to higher loyalties” responds more strongly to classroom justice than “condemnation of condemners,” with its mediation effect being nearly four times stronger than the latter. This indicates that students process perceived injustice more through redefining behavioral value rather than directly attacking authority, providing new theoretical insights for the differentiated application of neutralization theory across different contexts.

### 5.2 Practical implications

This research provides some insights for educational practitioners to understand the causes of student cyberloafing and to take some precautions. We recommend that teachers adopt specific measures to enhance classroom justice. In terms of distributive justice, teachers should establish transparent grading criteria by clearly explaining to students at the beginning of the course the specific weightings and grading details for class participation, assignments, midterm exams, and final exams. They should ensure equal participation opportunities by establishing a rotation speaking system, combining random calling with voluntary hand-raising to avoid always having the same students answer questions, while implementing anonymous grading for important assignments and exams to reduce the influence of subjective bias. In terms of procedural justice, teachers can jointly establish classroom rules with students by organizing discussions at the beginning of the semester, allowing students to participate in formulating rules regarding classroom discipline, assignment submission, and leave policies. They should establish transparent decision-making mechanisms by providing advance notice and soliciting student opinions for major instructional adjustments, and develop clear disciplinary procedures to ensure consistency in handling processes. In terms of interactional justice, teachers should improve their communication approaches by adopting “behavior-oriented” expressions rather than “personality-oriented” attacks when providing criticism, establish fixed teacher-student communication time by setting weekly office hours to accept student consultations and feedback, and provide timely and specific positive feedback for student progress (Baniasadi et al., 2023). Even though it is difficult for teachers to ensure complete fairness in the classroom, making students feel that their teachers value fairness in the classroom through these initiatives alone can work extremely well (Tierney, 2014).

Furthermore, this study’s findings regarding the mechanisms of neutralization techniques help teachers understand the cognitive processes underlying students’ cyberloafing behaviors. To address the “condemnation of condemners” technique, teachers can build positive teacher-student relationships by frequently using students’ names in classroom interactions, implement fair reward and punishment systems by applying uniform standards for handling disciplinary violations and providing timely praise for excellent performance, and establish appeal channels for students regarding instructional management decisions to ensure students have legitimate avenues for expressing dissatisfaction. To address the “appeal to higher loyalties” technique, teachers should enhance teaching quality by adopting highly interactive teaching methods such as case-based instruction and flipped classrooms to increase classroom attractiveness, and design meaningful learning tasks by connecting course content with students’ future career development and current social issues. It is worth mentioning that there are always some students who can find one or the other thing to criticize and complain about the classroom and they want to force the teacher to relax the classroom supervision so that they can do whatever they want in the classroom (Sharma, 2020). In this regard, they expect teachers to stick to their duties and coercive measures and harsh punishments are not a bad remedy (Kwahk et al., 2018).

### 5.3 Limitations and future research

It is necessary for us to elaborate on some limitations of this study. First, the development of neutralization theory has extended to dozens of neutralization techniques, and it is difficult to test all of them in our current study due to the content overlap and complexity that exists between different neutralization techniques, as well as the large size of the project. Future research should screen out more neutralization techniques that might work and test these neutralizations one at a time. Second, in terms of data collection, although our study collected samples at two points in time, our measures of students’ perceived classroom justice and intention to cyberloaf relied on students’ self-reports rather than actual observation. Our questionnaire was anonymous, but students may have underestimated their intention to cyberloaf and their perceptions of injustice for ethical reasons. We suggest that it is necessary for future studies to add teachers’ assessment of students’ cyberloafing behavior to the questionnaire, and to further test the findings through experimental methods. In addition, the samples we collected were mainly from students in some universities in East Central China, and the sample size was limited; we encourage researchers to collect more data from schools in different regions for the study.

For future research, we offer some directions for reference based on this research. First, as elaborated above, there are a variety of neutralization techniques, and neutralization techniques such as denial of responsibility, metaphors of ledgers, and denial of harm have all been found to be associated with cyberloafing (Cheng et al., 2014; Sharma, 2020). Future research could extend the structure of the present model to test the role that more neutralizing techniques play between classroom justice and intention to cyberloaf, and analyze the reasons why they do or do not play a role. In addition, individual factors such as self-efficacy, responsibility, and achievement orientation have been found to moderate the relationship between self-control and cyberloafing, and these factors may have the same qualities that enhance or inhibit the relationship between classroom justice and cyberloafing intentions (Prasad et al., 2010; Zhou et al., 2021). Existing studies have shown that neutralization techniques are significantly associated with cyberloafing, except for classroom justice, habituation, addiction, social support, and interference with peers’ cyberloafing behavior, which significantly predict students’ cyberloafing behavior (Sevinç and Dogusoy, 2022; She et al., 2025). Scholars can examine whether the mediating role played by neutralization techniques between these antecedent variables and cyberloafing holds true.

## 6 Conclusion

This study sought to determine whether students’ perceived classroom justice can have an impact on their intention to cyberloaf in the classroom, as well as the mediating pathways involved, with the aim of providing new perspectives on strategies to curb students’ cyberloafing in the classroom. The results suggest that classroom justice is negatively related to students’ intention to cyberloaf.; two neutralization techniques, condemning the condemned and appealing to higher loyalties, play a mediating role between classroom justice and intention to cyberloaf. Thus, students’ intention to cyberloaf can be reduced by improving classroom justice. Educators can use the questions in Chory’s Classroom Justice Scale to focus on justice in terms of distribution, procedure, and interaction. In addition, by focusing on whether classroom benefits are commensurate with the time and effort students put into the classroom, and by improving the quality of content so that students feel they are getting “value for money,” they can demonstrate greater loyalty to the classroom.

## Data Availability

The raw data supporting the conclusions of this article will be made available by the authors, without undue reservation.
